# Increased Connexin36 Phosphorylation in AII Amacrine Cell Coupling of the Mouse Myopic Retina

**DOI:** 10.3389/fncel.2020.00124

**Published:** 2020-06-01

**Authors:** Seema Banerjee, Qin Wang, Fuxin Zhao, George Tang, Chunghim So, Dennis Tse, Chi-Ho To, Yun Feng, Xiangtian Zhou, Feng Pan

**Affiliations:** ^1^Centre for Myopia Research, School of Optometry, The Hong Kong Polytechnic University, Kowloon, Hong Kong; ^2^School of Optometry and Ophthalmology and Eye Hospital, Wenzhou Medical University, The State Key Laboratory of Optometry, Ophthalmology and Vision Science, Wenzhou, China; ^3^School of Clinical Medicine, University of Cambridge, Addenbrooke’s Hospital, Cambridge, United Kingdom; ^4^Department of Ophthalmology, Peking University Third Hospital, Beijing, China

**Keywords:** amacrine cell, ganglion cell, myopia, retina, gap junction (connexin)

## Abstract

Myopia is a substantial public health problem worldwide. In the myopic retina, distant images are focused in front of the photoreceptors. The cells and mechanisms for retinal signaling that account either for emmetropization (i.e., normal refraction) or for refractive errors have remained elusive. Gap junctions play a key component in enhancement of signal transmission in visual pathways. AII amacrine cells (ACs), coupled by connexin36, segregate signals into ON and OFF pathways. Coupling between AII ACs is actively modulated through phosphorylation at serine 293 via dopamine in the mouse retina. In this study, form deprivation mouse myopia models were used to evaluate the expression patterns of connexin36-positive plaques (structural assay) and the state of connexin36 phosphorylation (functional assay) in AII ACs, which was green fluorescent protein-expressing in the Fam81a mouse line. Single-cell RNA sequencing showed dopaminergic synapse and gap junction pathways of AII ACs were downregulated in the myopic retina, although Gjd2 mRNA expression remained the same. Compared with the normal refractive eye, phosphorylation of connexin36 was increased in the myopic retina, but expression of connexin36 remained unchanged. This increased phosphorylation of Cx36 could indicate increased functional gap junction coupling of AII ACs in the myopic retina, a possible adaptation to adjust to the altered noisy signaling status.

## Introduction

Myopia (near-sightedness) is the most common refractive error (RE). In myopia, the eye is relatively long for the optical power of the cornea and lens resulting in distant images focusing in front of the photoreceptors (Rabbetts, 2007). As a growing public health problem, myopia affects more than 40% of individuals older than 12 years in the United States (Vitale et al., 2009) and more than 80% of people in Hong Kong (Morgan et al., 2012; Cho and Tan, 2018). Emmetropization is the term for the active process, which causes the expanding eye length to match the power of the cornea and lens resulting in correct focusing of distant images on the retinal photoreceptors. Refraction problems, such as myopia, occur if emmetropization does not proceed correctly.

There is ample evidence to suggest that the retina contains mechanisms to sense the focus of images and then generates signals to regulate eye growth during refractive development. The induction of form-deprivation myopia (FDM) by goggle wear or lid suture in chick (Schaeffel and Feldkaemper, 2015), mouse (Jiang et al., 2018), monkey (Hung et al., 1995), and marmoset (Graham and Judge, 1999) models has demonstrated that the retina plays a key role in eye growth control.

Gap junctions play important functional roles, such as signal averaging, noise reduction (DeVries et al., 2002), and neuronal synchronization (Deans et al., 2001), to code fundamental visual information in the retinal circuit. Modulation of gap junctions contributes to retinal plasticity, which enables the retina to adapt to visual inputs as self-adjusting neuronal networks (O’Brien, 2014; O’Brien and Bloomfield, 2018). The AII amacrine cells (ACs), coupled by Cx36 gap junctions (Feigenspan et al., 2001; Mills et al., 2001; Kihara et al., 2009) are the most numerous AC type in the mammalian retina. Cx36 is predominantly expressed by gap junctions between AII/AII ACs and AII/bipolar cells (Feigenspan et al., 2001) and is thought to be required for gap junctional coupling of most ganglion cell subtypes in the mouse retina (Pan et al., 2010). Heterologous gap junctions (Cx36–Cx45) between AII AC/ON-cone bipolar cell are found in strata 3 and 4 (S3 and S4) of the inner plexiform layer (IPL), whereas the homologous gap junctions (Cx36–Cx36) between AII ACs are located in the innermost part S5 of the IPL (Kolb, 1979; Sterling, 1983; Strettoi et al., 1990; Strettoi et al., 1992; Chun et al., 1993; Hartveit and Veruki, 2012). In this study, Ser293 labeling and Cx36 labeling were observed in the S5 layer where AII ACs are connected to each other.

AII ACs gap junctional coupling has high plasticity and is modulated through phosphorylation at serine110 and 276/293 (Ser 276 in teleost fish, Ser 293 in mammals) (Kothmann et al., 2009, 2012; Meyer et al., 2014). Dopamine actively modulates the Cx36 gap junction in AII ACs through activation of protein kinase α (PKA) (Kothmann et al., 2009). Both dopamine and dopamine D_1_ receptors play key roles in myopia development in the mouse retina (Zhou et al., 2017; Huang et al., 2018). However, which cell types and neurotransmitters are affected in the myopic retina and how they adapt to defocused status remain unknown.

Single-cell RNA sequencing (scRNA-seq) has been applied to determine the functional implications of each cell type in the retina via their gene expression (Macosko et al., 2015). In this study, results of scRNA-seq uncovered a dopaminergic synapse pathway with distinct gene-expression profiles that accounts for the cellular phenotypic changes during myopia pathogenesis. But this is not the case for gap junction delta-2 protein [*GJD2*, also known *as* connexin36 (Cx36)]. Therefore, it is possible that the phosphorylation state of Ser293, indicative of the function of coupling through Cx35/36 gap junctions affected by the dopamine pathway, would increase in the myopic retina.

To test this hypothesis, the expression patterns of Cx36-positive plaques (structural assay) and the state of Cx36 phosphorylation (functional assay) in AII ACs in a mouse model of myopia were evaluated by using specific antibodies to the phosphorylated form of Cx36. It was found that phosphorylation of Cx36 gap junctions in AII ACs increased in the mouse myopic retina. At the same time, expression of Cx36 remained unchanged. The increased phosphorylation of Cx36 may indicate increased functional gap junction coupling of AII ACs in the myopic retina to filter out the noise signaling in defocused images. Understanding the regulation of Cx36 function is important to understand the visual signaling processes in both the normal and myopic retina.

## Materials and Methods

### Ethics Statement

All animal procedures were approved by the Animal Subjects Ethics Sub-Committee of the Hong Kong Polytechnic University and the Animal Care and Ethics Committee at Wenzhou Medical University (Wenzhou, Zhejiang, China). All experiments complied with the Guide for the Care and Use of Laboratory Animals published by the National Institutes of Health.

### Animals

As in the human retina (Jonas et al., 1992), rods constitute 97% of mouse retinal photoreceptors (Carter-Dawson and LaVail, 1979). Adult mice (postnatal days 16–56) C57BL/6J (RRID:IMSR_JAX:000664) wild-type (WT), *n* = 51, and Fam 81a [Mouse Genome Informatics (MGI):1924136, postnatal days 48–56] from MGI, *n* = 7 of either sex, were used in the study. Fam 81a mice have green fluorescent protein (GFP)-labeled AII ACs, which were used for Neurobiotin injection. Cx36^–/–^ knockout mice (RRID:MGI:3810172) first generated in David Paul’s laboratory, Harvard Medical School, were a kind gift from Samuel M. Wu, Baylor College of Medicine (*n* = 4).

The form deprivation method for inducing experimental mouse myopia (lid suture) was used (Barathi et al., 2008): right eyes (OD) were sutured with 7° nylon non-absorbable sutures, black monofilament (Alcon Surgical, Fort Worth, TX, United States), at postnatal day 16 (lid suture after visual experience) (Tejedor and de la Villa, 2003). The unsutured left eyes (OS) served as experimental controls. Suturing was checked every day, and if the knots had loosened, the eyelids were resutured immediately. Suturing was continued for 40 days, being removed on postnatal day 56. Refraction and axial length (AL) measurements were performed on the same day, followed by processing of the retinas. All immunology experiments were performed on light-adapted animals in the daytime phase of their light cycle (Kothmann et al., 2009; Ivanova et al., 2015).

### Refraction Measurements in Mouse Model

An infrared photorefractor (Steinbeis Transfer Center, Stuttgart, Germany) (Schaeffel, 2008) was used to measure the refractive state. To decrease movement of the mice and measure the REs accurately, they were lightly anesthetized with an intraperitoneal injection of ketamine (Vedno, St. Joseph, MO, United States) and xylazine (Akorn, Decatur, IL, United States) [10 and 1 mg (kg body weight) ^–1^, respectively]. The eyes were instilled with tropicamide, phenylephrine hydrochloride solution (Mydrin-P ophthalmic solution; Santen Pharmaceutical Co., Ltd., Osaka, Japan), 5 min before the measurement to ensure mydriasis and cycloplegia. To determine refraction, 20 measurements were taken along the optical axis for each eye, and the averages were calculated. The results were confirmed by streak retinoscopy refraction. If a difference of refractive power greater than 5 diopters (D) between the two methods was observed, the animal was excluded.

The infrared photorefractor was also used to measure the corneal curvature (Schaeffel, 2008), which was determined by the diameter of the circle made by the reflected light on the mouse cornea. The aim was to rule out corneal damage in the lid-suture mouse model.

### Axial Length Measurement in Mouse

Axial length of the mice was measured with spectral domain–optical coherence tomography (SD-OCT) under light anesthesia. The anesthetized mouse was placed in a cylindrical holder, which was attached to an X-Y-Z movable stage (Bioptigen Spectral Domain Ophthalmic Imaging System, Envisu R4410 SD-OCT, Leica Microsys-tems, Wetzlar, Germany) in front of the SD-OCT light source. The cornea was hydrated with normal saline. The reference arm and focus dial were adjusted simultaneously to a point at which all structures of the eye were in focus. Correct alignment was confirmed by viewing the radial image of the surface of the eye and adjusting the light source for the central reflection along the horizontal and vertical optical meridians. Each scan contained an average of 10 images. To measure AL, calipers (calibrated at refractive index of 1.38) were placed from the cornea to lens fold and retinal pigment epithelium (RPE) border to lens fold.

### Flattened Retina–Sclera Preparation

All experiments were performed during daylight hours. The mice were anesthetized deeply with an intraperitoneal injection of ketamine and xylazine [80 and 10 mg (kg body weight) ^–1^, respectively], and lidocaine hydrochloride (20 mg mL^–1^) was applied locally to the eyelids and surrounding tissue. Eyes were removed under dim red illumination and hemisected anterior to the ora serrata. The anterior optical structures and the vitreous humor were removed, and the resultant retina–eyecup with sclera attached, either whole or in sections, was placed in a super-fusion chamber. For patch recordings, retinas were dissected into four equal quadrants and attached to a modified translucent Millicell filter ring (Millipore, Bedford, MA, United States). The flattened retinas were superfused with oxygenated mammalian Ringer solution, pH 7.4, at 32°C (Bloomfield and Miller, 1982). The anesthetized animals were killed by cervical dislocation immediately after enucleation.

### Electrical Recording

Extracellular recordings were obtained from retinal ganglion cells (RGCs) (eight WT mice) in all retinal quadrants. Whole-cell recordings were performed by using an Axopatch 700B amplifier connected to a Digidata 1550B interface and pCLAMP 10 software (Molecular Devices, San Jose, CA, United States). Cells were visualized with near-infrared light (>775 nm) at 40× magnification with a Nuvicon tube camera (Dage-MTI, Michigan, IN, United States) and differential interference optics on a fixed-stage microscope (Eclipse FN1; Nikon, Tokyo, Japan). Retinas were superfused at a rate of 1 to 1.5 mL min^–1^ with Ringer solution, composed of (in mM) 120 NaCl, 2.5 KCl, 25 NaHCO_3_,0.8 Na_2_HPO_4_, 0.1 NaH_2_PO_4_, 1 MgCl_2_,2 CaCl_2_, and 5 D-glucose. The bath solution was continuously bubbled with 95% O_2_–5% CO_2_ at 32°C (Pan et al., 2016).

Electrodes were pulled to 5 to 7 MΩ resistance, with internal solutions consisting of (in mM) 120 potassium gluconate, 12 KCl, 1 MgCl_2_, 5 EGTA, 0.5 CaCl_2_, and 10 HEPES (pH adjusted to 7.4 with KOH). This internal solution was used in experiments in which spiking was not blocked. Spike trains were recorded digitally at a sampling rate of 10 kHz with Axoscope software, which were sorted by using Off-line Sorter (Plexon, Dallas, TX, United States) and NeuroExplorer (Nex Technologies, Littleton, MA, United States) software.

### Injection of Neurobiotin

The GFP-labeled cells of FAM81a were visualized at 40× magnification, as described above, and were impaled under visual control using pipette tips filled with 4% Neurobiotin (Vector Laboratories, Burlingame, CA, United States) and 0.5% Lucifer Yellow-CH (Molecular Probes, Eugene, OR, United States) in double-distilled water and then back filled with 3M KCl. The electrode resistance was ∼100 MΩ. The impaled cells were then injected with a biphasic current (+1.0 nA, 3 Hz). For pharmacology experiments of AII ACs, injections were performed for 1 min followed by a 10-min diffusion period. Each retina piece was superfused, starting 15 min prior to the start of the injection and on through the diffusion period, with either bubbled Ringer solution (at 35°C) or with dopamine receptor (D_1_R) agonist (SKF38393, SKF38393 [(±)-1-phenyl-2,3,4,5-tetrahydro-(1H)-3-benzazepine-7,8-diol hydrobromide], 10 μM; Tocris, Ellisville, MO, United States) or antagonist (SCH23390, SCH23390 [*R*(+)-7-chloro-8-hydroxy-3-methyl-1-phenyl-2,3,4,5-tetrahydro-1*H*-3-benzazepine hydrochloride] at low concentrations 5 μM, Sigma-Aldrich, St. Louis, MO, United States; D-054). The pharmacology experiments were performed under dim white room lights, with approximately 1.27 × 10^3^ photons μm^–2^ s^–1^ measured at the location. After the injection, the retinal pieces were fixed in 4% paraformaldehyde for at least 10 min. The tissues were incubated overnight at 4°C in 0.1 M phosphate buffer (PB) with 0.5% Triton-X 100 and 0.1% NaN_3_ containing 1% donkey serum and then, after extensive washing, incubated in Alexa-488-conjugated streptavidin (Invitrogen, Carlsbad, CA, United States) 1:200 overnight at 4°C. The tissues were then mounted in Vectashield (Vector Laboratories) for observation.

### Patterned Light Stimulation

A green organic light-emitting display (OLED, Olightek, China; 800 × 600-pixel resolution, 85 Hz refresh rate) was controlled by an Intel Core Duo computer with a Windows 7 operating system. In this setup, using a Nikon 40x water-immersion objective (CFI Apo 40XW NIR, NA = 0.8), the area of the retina that received light stimuli was 250 μm in diameter. Under the 40× objective, the 15 μm diameter pixels of the OLED were 0.25 μm/pixel on the retina. Spatial frequency stimuli were generated by PsychoPy onto the photoreceptor layer. The background light intensity was ∼700 isomerizations Rh^∗^/rod/s and the stimulus was ∼1.816 × 10^5^ Rh^∗^/rod/s. At this level of background illumination, the rod pathway has been shown to be saturated, leaving the cone pathway to mediate the light response (Borghuis et al., 2013). The detailed description of the patterned light system generating defocused images on retina can be found in the Supplementary Material provide with a previously published paper (Pan, 2019).

### Immunocytochemistry

#### Antibodies

Mouse anti-Cx35/36 (mCx36, 1:1,000, EMD Millipore Cat# MAB3045, RRID:AB_94632) was used for mouse retina and mouse polyclonal antibody anti-Cx36 (1:1,000; Invitrogen; catalog no. 36-4600, RRID:AB_2533260) for chicken retina labeling (Kihara et al., 2009; Ivanova et al., 2015). Rabbit anti-Ser293-P (Ser293-P, 1:1,000, kindly provided by Dr. John O’Brien, The University of Texas) was used for retinal labeling (Kothmann et al., 2007). Monoclonal mCx35/36 and polyclonal antibody anti-Cx36 were verified by Western blot in both mouse and chicken retinas (Kothmann et al., 2007). Goat anti-ChAT (1:500; Millipore; Cat# AB144P, RRID:AB_2079751) was used for mouse retina to label the ON and OFF layers in the IPL.

For the mouse retina, samples were obtained from the dorsal section of the midperipheral retina in the nasotemporal plane. The retinal pieces, attached with filter paper (RGCs up) after being isolated from eyecups, were submersion-fixed in 2% *N*-(3-dimethylaminopropyl)-*N*’-ethylcarbodiimide hydrochloride (“carbodiimide”; Sigma-Aldrich) in 0.1 M PB, pH 7.5, for 30 min at room temperature. After fixation, the retinas were separated from the filter paper and washed with PB extensively with 0.1 M PB (pH 7.4) and blocked with 3% donkey serum in 0.1 M PB with 0.5% Triton-X 100 and 0.1% NaN_3_ overnight. The antibodies were diluted in 0.1 M PB with 0.5% Triton-X 100 and 0.1% NaN_3_, containing 1% donkey serum. The tissues were incubated in primary antibodies for 3 to 7 days at 4°C and, after extensive washing, incubated in secondary antibodies overnight at 4°C. After washing with 0.1 M PB, the tissues were mounted in Vectashield (Vector Laboratories) for observation.

#### Data Acquisition and Analysis

Retinal whole mounts were acquired on a Zeiss LSM 800 with an Airyscan (Zeiss, Thornwood, NY, United States) confocal microscope using a 63× objective (NA 1.4). The XY resolutions of the instrument were 120 and 350 nm in z resolution, and all three channels were superimposed. *Z*-axis steps were usually 0.35 μm. The size threshold was filtered by 0.01 μm^2^. The retinas from the mice were imaged under identical acquisition conditions, including laser intensity, pinhole, photomultiplier amplification, and z-stack step size. Three animals from each mouse line were examined. The analysis was conducted as described previously (Kothmann et al., 2009, 2012; Ivanova et al., 2015). Four fields were examined for each mouse retina, and the images processed and analyzed by using ImageJ software (ImageJ, An open source developed by National Institutes of Health, Bethesda, MD, United States, 1.52i, RRID:nif-0000-30467). The resulting data are presented as mean ± standard error unless otherwise indicated. The ratio of the mean intensity of Ser293-P to mCx36 immunofluorescence was calculated for each of the regions of interest (ROIs) and averaged across all ROIs in all images per condition. In this way, it was possible to collapse the phosphorylation data into one value per condition per animal to perform statistical analysis.

Statistical analyses were performed by using Origin software (OriginLab, Northampton, MA, United States) and SPSS version 25 (Armonk, NY, United States). Statistically significant differences (*P* < 0.05) were determined by using Student *t*-test and Wilcoxon signed ranks test in Figure 7.

### Retinal Single-Cell Suspensions

For the FDM mouse model, 21-day-old C57BL/6J mice with diffuser goggles were used (Schaeffel et al., 2004; Wu et al., 2018). After 2 days’ form deprivation treatment, the mice were sacrificed by cervical dislocation, with their eyeball enucleated and dissected to obtain the whole retina. The retinas were digested in collagenase IV (6 mg/mL; Gibco BRL, Grand Island, NY, United States) for 1 min, before moving the RPE layer. Digestion in papain solution (10 mg/mL Dulbecco phosphate-buffered saline (DPBS; Sigma-Aldrich) of remaining retina was performed for a further 30 to 45 min. Papain was then neutralized with a trypsin inhibitor solution (0.15% ovomucoid in DPBS; Gibco BRL), and the tissue was triturated with 200-μL pipette tips to generate a single-cell suspension. The cells were pelleted, resuspended, and filtered through a 40-μm nylon cell strainer (Corning Inc., Corning, NY, United States) to eliminate all clumped cells. The cells were then diluted in DPBS to ∼1,000 cells/μL for use.

### Single-Cell RNA Sequencing

Single-cell sequencing was carried out at Beijing CapitalBio Technology Co., Ltd., Beijing, China. Six independent retina samples (three form-deprived treated eyes and three fellow eyes) were captured (three lanes for each sample), making a total of 18 batches by using the 10X Chromium system (10X Genomics). The cells were partitioned into Gel Bead-in-Emulsions and barcoded cDNA libraries and then prepared for analysis by using the single-cell 30 mRNA kit (V2; 10X Genomics). Single-cell libraries were sequenced in 150-bp paired-end configuration by using an Illumina, San Diego, CA, United States HiSeq X Ten. The Seurat R/Bioconductor toolkit was used to perform the dimension reduction process. (The photoreceptor was removed manually based on the existing markers). The top 30 principal components were ultimately used in the RunTSNE analysis. Gene expression levels were quantified as CPM (counts per million) based on count data (Subramanian et al., 2005).

### GSEA Analysis

Genes were ranked according to changes of average expressed value and difference of percentage of expressed cells between fellow and treated groups to generate rank files, which were used as input for the GSEAPreranked module of the GSEA v2.0 tool. The pathways of dopaminergic synapse and gap junction were directly downloaded from the KEGG (Kyoto Encyclopedia of Genes and Genomes) database (Kanehisa, 2019; Kanehisa and Goto, 2000).

## Results

### Single-Cell RNA Sequencing Showed That the Dopaminergic Synapse and Gap Junction Pathways Were Downregulated in the Myopic Retina, but Gjd2 mRNA Expression in AII ACs Was Unaltered

For single-cell sequencing data, after filtering out cells with fewer than 100 genes and more than 5,000 genes, the remaining cells were used for t-distributed stochastic neighbor embedding (tSNE) analysis. A total of 26 distinct cell clusters were identified based on tSNE. Slc6a9 was used to identify the glycinergic amacrine. Gjd2 highly expressed clusters in the glycinergic amacrine were defined as the AII ACs. A density clustering approach was combined with *post hoc* differential expression analysis to divide the cells into transcriptionally distinct clusters.

**FIGURE 1:**
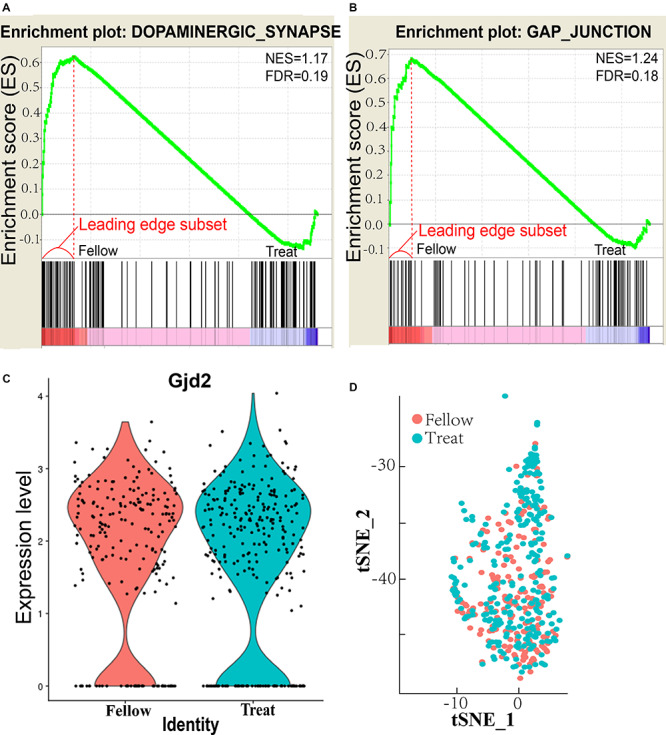
Enrichment pathways and plots of AII amacrine cells (ACs). **(A)** Enrichment plot of dopaminergic synapse. NES, normalized enrichment score; FDR, false discovery rate *q* value, <0.25 is statistically significant. **(B)** Enrichment plot of gap junction. **(C)** Retinal Gjd2 mRNA expression in treated eyes and fellow eyes after form deprivation for 2 days, no significant difference exists. **(D)** tSNE analysis of AII AC clusters in treated and follow eyes showed that there was no significant difference in cell numbers between treated and follow eyes.

AII–AII AC coupling is regulated by dopamine (Kothmann et al., 2009), and 25% of AII ACs in the mouse retina express D_1_Rs (Farshi et al., 2016). The dopaminergic synapse in the AII AC pathway is downregulated in the form deprivation-induced myopic retina (Figure 1A). The normalized enrichment score (NES) = 1.17 and false discovery rate (FDR) *q* value = 0.19 both differed significantly from those of the untreated fellow eye (<0.25 for FDR is considered as significantly different). The leading edge subset-associated genes in the dopaminergic synapse pathway are shown in Supplementary Figure S1 and Supplementary Table S1. D_1_ receptor downregulated pathway genes included Gαq and phospholipase C (PLC) (Lee et al., 2014), inositol trisphosphate receptor (IP3R), CREB (cAMP response element-binding protein), and protein phosphatase 2A (PP2A), all of which are involved in the dopamine–PKA system (Undieh, 2010). This suggests that the dopamine level was decreased in AII ACs in the myopic retina. Furthermore, it also indicates that synthesis and secretion of dopamine were decreased, and the dopaminergic synapse pathway was downregulated.

The gap junction pathway of the AII ACs was also downregulated in the myopic retina (Figure 1B), NES = 1.24 and FDR *q* value = 0.18, which were significantly different from the untreated fellow eye. The leading edge subset-associated genes involved in the gap junction pathway are shown in Supplementary Figure S2 and Supplementary Table S1. The gap junction pathway involved the genes shown in Supplementary Figure S2. These genes included Gαq and PLC, which were the same in the D_1_ receptor downregulated pathway.

However, expression of the gap junction delta-2 protein (*Gjd2*, also known as Cx36) in the AII ACs did not differ significantly between the induced myopic eyes and the fellow eyes (Figure 1C). The result showed that expression of Cx36 was not affected in the myopic retina. The function of Cx36 will be investigated in future studies. For at the II ACs cluster, tSNE analysis showed that there was no significant difference in cell numbers of AII ACs between treated and fellow eyes (χ^2^ test, *P* = 0.057; Figure 1D). The results of tSNE analysis indicate that there was no change in FDR numbers of AII AC cells in myopia induction in the mouse retina.

In summary, RNA sequencing showed that both the dopaminergic synapse pathway and the gap junction pathways are downregulated in AII ACs of the myopic retina, but Gjd2 mRNA expression remained the same. The result may indicate that dopamine levels and the gap junction were influenced in coupling of AII–AII ACs, resulting in abnormal signal transduction and visual processing in the form deprivation-induced myopic retina.

### Measurement of REs by Photorefraction and OCT

**FIGURE 2:**
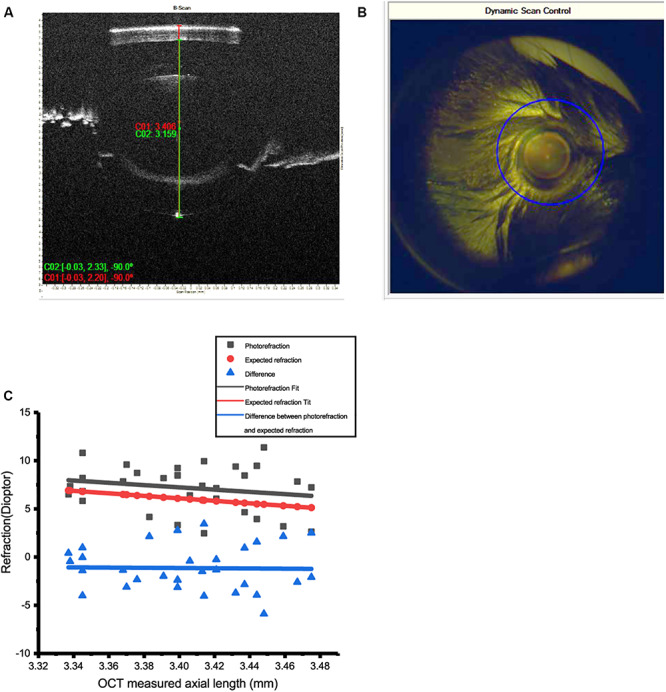
Measurement of refractive errors (RE) in mice by photorefraction and OCT. **(A)** Axial length (AL) of the mouse eye was measured from the surface of the cornea to the RPE layer by OCT. **(B)** The optical axis of the mouse eye can be precisely aligned with dynamic scan control of OCT. **(C)** AL measured by OCT could be used to predict RE in 8-week-old WT mice. Refractive errors of 30 eyes of WT mice (photorefraction) fitted by linear regression (red line, *R*^2^ = 0.042) matched the data of AL estimated from OCT (black line, *R*^2^ = 0.99). The difference between these two methods is close to 0 (blue line, *R*^2^ = 0.1%). Calculation: RE = 38 – 1.544/(1.03 × AL).

Refractive error of the mice was measured by infrared photorefraction on postnatal day 56, averaged 7.18 ± 0.42 D (mean ± SEM, *n* = 32). Twenty measurements were acquired for each eye to minimize the error. Conventional streak retinoscopy was also used to measure the RE. Axial length, measured with OCT at 8 weeks, was 3.40 ± 0.007 mm (mean ± SEM, *n* = 32) (Figures 2A,B).

An infrared photorefractor can acquire relatively accurate measurements of RE with a dilated pupil. However, as variation existed between eyes, calculating RE from AL measured by OCT can provide more accurate and consistent data.

The RE index of mouse eyes at 6 to 10 weeks has previously been determined as 1.544 (Remtulla and Hallett, 1985; Schmucker and Schaeffel, 2004). Linear regression was performed to demonstrate that the REs of WT mice eyes matched the calculation from AL by OCT (*n* = 38). Refractive errors of 32 eyes at 8 weeks showed a good linear regression fit with the calculation of AL by OCT. Therefore, optical modeling of the mouse myopic eyes used to establish the RE was calculated by the following equation in 8-week WT mice.

$$R\,E=38-\frac{1.544}{1.03\times A\,L}$$

The mean RE of mice was 7.18 D at postnatal day 56 (Figure 2C), similar to those of other studies (Schmucker and Schaeffel, 2004; Schaeffel, 2008). If, as previously determined (Schmucker and Schaeffel, 2004), an increase in AL of 5 μm induced equal to 1-D refractive power in mouse eye, then 100 μm defocus could induce ±20-D RE.

The AL of form deprivation-induced myopic eyes (eyelid sutured after 40 days) was 3.71 ± 0.08 mm, in contrast to 3.60 ± 0.08 mm (mean ± SD, *P* < 0.05, *n* = 11) of the contralateral control eyes (an average increase in AL of 0.11 ± 0.05 mm after lid suturing). This increase is estimated to be equal to 20-D defocus.

The RE of mice after lid suturing varied from +9 to −10 D. Those with a RE of less than −5 D, confirmed by both OCT and infrared photo refractor measurement, were selected as mouse model of myopia.

### Defocused Images in the Myopic Retina Changed Spike Properties of Ganglion Cells, Which Code Visual Signaling

More than 30 types of RGCs exist in the mouse retina to detect and code distinctive aspects of visual information and transmit this to the brain (Badea and Nathans, 2004; Coombs et al., 2006; Volgyi et al., 2009). In the myopic retina, a defocused image changed the signaling of RGCs as observed in the previous studies (Pan, 2019).

**FIGURE 3:**
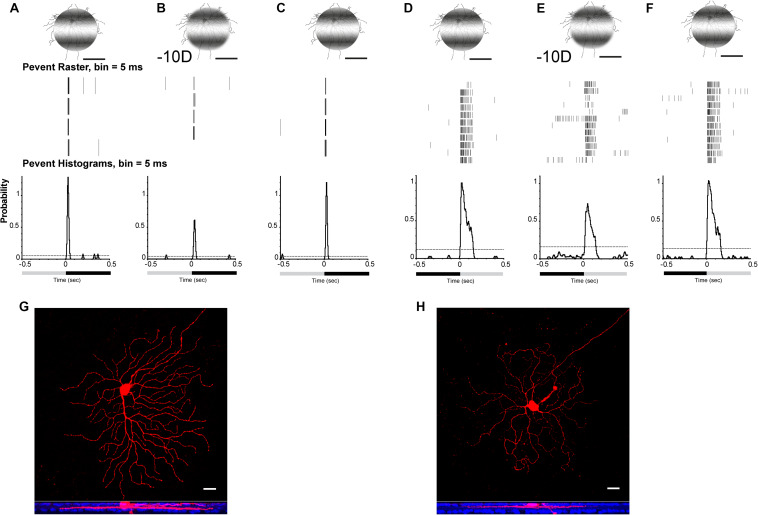
Defocused images altered the signaling responses in RGCs. Perievent rasters histogram of OFF **(A–C)** α-RGC and ON **(D–F)** α-RGC responses (middle images) to 0.5-s flashed 0.002 cycles/degree light stimuli. Light spot was maintained in 125 μm when defocused –10 D **(B,E)**, whereas light intensity decreased from I = 5.1 × 10^4^ to 5.0 × 10^4^R*/rod/s. OFF α-RGC light responses decreased after a defocused image was projected **(B)**. Peristimulus time histogram of the OFF α-RGC showed that the probability of spikes decreased from 1.28 to 0.61 (–10-D defocus), then back to 1.21 after refocusing. ON α-RGC **(D)** decreased from 1.05 to 0.7 (–10-D defocus) and then back to 1.05 after refocusing. **(G)** and **(H)** showed OFF α-RGC **(A–C)** and ON α-RGC **(D–F)** with Neurobiotin filling after recording. Lower images show the ganglion cell branched in OFF and ON layer with anti-ChAT labeling (blue). Scale bar: **(A–F)** 75 μm, **(G,H)** 20 μm.

Projection of optical defocused images was performed by custom-made patterned illumination (Pan, 2019). A 125-μm diameter, 0.002 cycles/degree flashed light (0.5 s) spot with light stimulus (I = 1.1 × 10^5^ R^∗^/rod/s) was projected on an ON or OFF α-RGC (Figures 3G,H). The image area projected on the RGC layer was adjusted by programming to render them identical (e.g., 125 μm in diameter) with respect to different defocus powers. Thus, the effect of a changed receptive field under the defocused images was minimized. The image was defocused 50 μm equal to −10-D RE induced in the mouse eye. The light intensity decreased from 5.1 × 10^4^ to 5.0 × 10^4^ R^∗^/rod/s, whereas the light spot projected on the RGC layer remained at 125 μm in diameter, but the edges became blurred.

The data from both ON (9 of 34, 38%) and OFF α-RGCs (12 of 22, 55%) showed that the probability of spikes decreased. The peristimulus rasters histogram of the OFF α-RGC showed that the probability of spikes decreased from 1.28 to 0.61 with defocused status (Figures 3A,B), whereas ON α-RGC decreased from 1.05 to 0.7 (−10-D defocus) (Figures 3D,E). Both the probability of spikes of ON and OFF α-RGC recovered after the image stimuli refocused on the photoreceptors (Figures 3C,F).

### Specificity of Anti-Cx36 Phosphorylation Antibodies in Mouse Retinas

Single-cell RNA sequencing revealed the dopaminergic synapse pathway was downgraded without gene expression change of Cx36. Therefore, it was hypothesized that the phosphorylation state of Ser293, indicator of the function of coupling through Cx35/36 gap junctions affected by the dopamine pathway, would change in the myopic retina.

**FIGURE 4:**
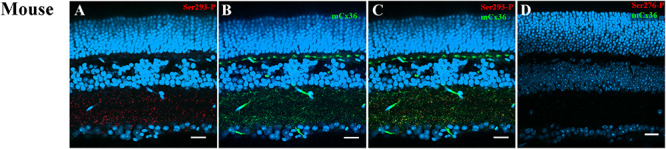
Ser293 antibody labeling patterns in mouse retina. **(A–C)** Labeling pattern of phospho-Ser293 antibody in vertical section of mouse retina. **(A)** Ser293-P antibody-labeled (red) abundant punctate structures in the inner plexiform layers (IPL) and some in the outer plexiform layer (OPL). **(B)** Labeling with monoclonal Cx36 antibody (green) shows the labeling of Cx36 also in the OPL and IPL. **(C)** Merged image of **(A)** and **(B)** shows multiple plaques identified as Ser-293-P colocalized with Cx36 antibody labeling (yellow). **(D)** In the negative control of Cx36 KO mouse retina, labeled punctate of Cx36 antibody and Ser293-P were absent in both OPL and IPL. Scale bar is 20 μm.

Ser293-P antibodies were used to visualize phosphorylated Cx36. This antibody has been previously tested in perch (Kothmann et al., 2007), rabbit (Kothmann et al., 2009, 2012), and mouse retinas (Ivanova et al., 2015). As previously reported (Ivanova et al., 2015), Ser293-P expression was clearly observed in the mouse IPL colocalized with the processes of AII ACs. In the current study, 95.4% ± 0.02% Ser293-P-positive puncta in the IPL were colocalized with mCx36-labeled plaques in the WT mouse retina (five retinas × three samples = 50,631 detectable in Ser293 phosphorylated plaques) (Figures 4A–C). The result confirmed that Ser293-P antibody was highly specific for Cx36 (97.8% ± 0.3%) as previously reported (Ivanova et al., 2015). Staining for both Cx36 and Ser293-P was consistently completely absent in OPL and IPL in the Cx36KO mouse retina (Figure 4D). The characteristic of the WT retina punctate of Cx36 and Ser293-P labeling, which disappeared in the Cx36 knockout mouse retina, confirms the high specificity of Cx36 and Ser293-P antibody labeling in the mouse retina.

### AII ACs in Mouse Retina and Their Colocalization With Connexin36

**FIGURE 5:**
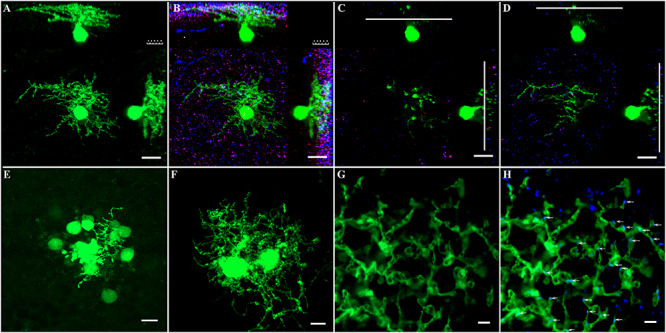
Phospho-Ser293 and monoclonal anti-Cx36 antibody labeled prominently in IPL. **(A)** GFP-labeled AII amacrine cell in Fam 81 mouse retina was targeted and injected with Neurobiotin to show single AII AC’s soma–dendritic morphology in Z stack by 3D reconstruction image. **(B)** AII AC double labeled with phospho-Ser293 (red) and monoclonal anti-Cx35/36Cx36 (blue) puncta located predominately on the arboreal dendrites (blue) strata three to five layers. **(C)** Single layer of AII AC in the lobular layer. **(D)** Single layer of AII AC in the arboreal dendrite layer. Phospho-Ser293 puncta are also colocated on the arboreal dendrites (red) strata layer 5. **(E)** Coupled AII ACs displayed with Neurobiotin injection. Only cell bodies coupled via gap junction showed clearly. **(F)** To show the dendritic gap junctional coupling in AII ACs, two nearby AII ACs were injected with Neurobiotin. **(G)** Dendrites between the two nearby AII ACs injected with Neurobiotin in the S5 layer. **(H)** Labeled with monoclonal anti-Cx35/36 (blue). Cx36 puncta localized on dendrites of AII ACs (white arrows). Scale bar: **(A–D)** 10 μm, **(E,F)** 5 μm, **(G,H)** 2 μm.

AII ACs have a bistratified structure. In the OFF sublamina layer, lobule dendrites of AII ACs contact with OFF bipolar cells via chemical synapses (Figure 5C). In the ON sublamina layer, the AII dendrites make gap junctions with other AII ACs and ON bipolar cells (Figure 5D). Far fewer Cx36 puncta were observed in the lobule’s dendrites layer, compared with that in the dendritic layer. Heterologous gap junctions (Cx36–Cx45) between AII AC/ON-cone bipolar cell gap junctions were found in S3 and S4 of the IPL, whereas the homologous gap junctions (Cx36–Cx36) between AII ACs located in the innermost part, S5 of the IPL (Figure 5H).

Dye injection can be used to show the coupling between AII–AII ACs; however, it is difficult to identify AII ACs in the mouse retina in the WT mouse. GFP labeling of AII ACs in mouse line-Fam81a allowed single AII AC to be visualized by Neurobiotin injection (Figure 5A). This was followed by double labeling of AII ACs with Cx36 and 293-P antibodies (Figure 5B). Most Cx36 puncta [96.7% ± 1.2% (SD)] were located within dendrites in ON sublamina. Ser-293 (93.7 ± 1.2%) puncta were also predominantly located in the same layer. The following experiments observed the phosphorylation state of Cx36 in AII ACs within the ON sublamina layer in the IPL. The S5 layer close to the RGC was observed in the following experiment (Figure 5D). The Cx36 puncta in S5 excluded the concern about mixed Cx36 puncta from AII AC and ON cone bipolar cell coupling.

AII AC coupling between soma and dendrites can be shown by Neurobiotin injection. However, the Neurobiotin filling predominately displayed soma via gap junctional coupling (Figure 5E). In order to show the dendritic gap junctional coupling, two neighboring AII ACs were injected with Neurobiotin (Figure 5F). Thus, the gap junctional coupling between the two AII ACs could be clearly observed with caution, especially in the dendritic layer of S5. Cx36 antibody labeling showed that the majority (97.7% ± 1.3%) of Cx36 puncta were colocated with dendrites of ACs in S5 (Figures 5G,H).

### Connexin36 Phosphorylation Changed in Myopic Mouse Retina

**FIGURE 6:**
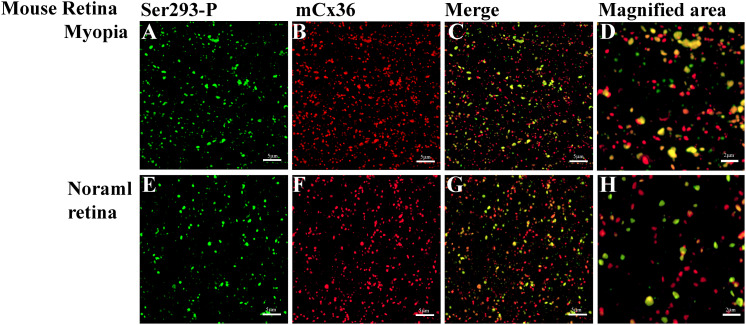
Phospho-Ser293 antibodies specifically recognizes Cx36 in the whole mount mouse retina. **(A–D)** Confocal stack sections in stratum 5 of the IPL of the myopic mouse retina: mCx36 labeled in red and its phosphorylated form, Ser293-P (green), are present with similar punctate labeling. The magnified areas show the merged images of phosphorylated Cx36, reflected by yellow color. **(E–H)** Phospho-Ser293 antibody recognizes Cx36 in the control mouse retina. Images are 2-μm deep stacks. Scale bar: **(A–C,E–G)** 5 μm, **(D,H)** 2 μm.

Cx36, present in mouse and rabbit retinas, which is homologous to Cx35 in perch, was recognized by the S293-P antibody (Figures 6A–H). Western blot had shown that Ser-293 antibodies were specific for the phosphorylated form of Cx35/36 (Kothmann et al., 2007). As the level of Cx36 phosphorylation differed in the myopic retinas from that in controls, this indicated that the functional state of gap junctions was altered in myopia. Because the expression level of Cx36 was also determined in the myopic retina, it is possible to compare the density of Cx36-positive plaques, their size, and the percentage of Ser-293 of Cx36 phosphorylation between the form deprivation-induced myopic retinas and untreated controls.

**FIGURE 7:**
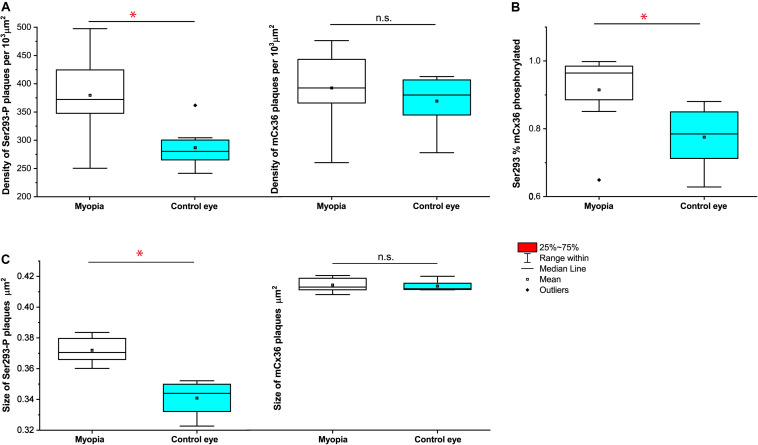
Quantification of phosphorylation of Cx36 gap junctions in AII amacrine cells in mouse myopic retinas. For S293, the density of phosphorylation reflected by detectable Ser293-P labeling **(A)**, the percentage of Ser293-P of Cx36 phosphorylated rate **(B),** and the size of Ser293-P plaque **(C)** were significantly increased in myopic retinas compared to controls. The density of mCx36 labeling **(A)** and the size of mCx36 plaque **(C)** did not differ between myopic and control mouse retinas. The data are presented as averages. Error bars are SEM. Significance is based on Wilcoxon signed ranks test, where *0.01 < *P* < 0.05, not statistically significant *P* > 0.05.

The quantification analyses of ser-293 P showed that (Figures 7A–C) a significant difference in the density of S293P plaques existed between WT [284 ± 12 per 10^3^ μm^2^ (means ± SEM), *n* = 8] and myopic mice (377 ± 27 per 10^3^μm^2^, *n* = 8, *p* = 0.017) (Figure 7A). The increase in density of S293P plaques in myopic retinas indicated more of Cx36 is phosphorylated compared to control. There was also a significant difference in the size of ser-293 P plaques between WT (WT 0.341 ± 0.003 μm^2^) and myopic retinas (0.372 ± 0.004 μm^2^, *p* = 0.012) (Figure 7C). The increase in size of S293P plaques in myopic retinas also suggested there was more labeled phosphorylation of S293 in Cx36 compared with control. A total of 14,773 individual plaques in eight myopic mice and 12,679 plaques in eight WT mice were analyzed and compared by Wilcoxon signed ranks test. In addition, there was difference (*p* = 0.036) in the percentage of Ser293 phosphorylated rate of Cx36 (Figure 7B). In WT mice, 77.5% ± 0.03% (means ± SEM, *n* = 8) of Cx36 plaques were phosphorylated, whereas in myopic retinas, 92.1% ± 0.04% (*n* = 8) of the plaque had detectable phosphorylation.

In contrast, there was no difference in the density of mCx36 plaques that existed between WT [369 ± 16 per 10^3^ μm^2^ (means ± SEM), *n* = 8] and myopic mice (392 ± 24 per 10^3^μm^2^, *n* = 8, *p* = 0.674) (Figure 7A). There was also no significant difference in the size of mCx36 plaques, with eight mice of 17,244 present in myopic retinas and eight mice of 14,516 in WT retinas (WT 0.412 ± 0.002 μm^2^; myopia 0.414 ± 0.003 μm^2^; *p* = 0.362) (Figure 7C). Each mouse line was represented by three different animals; each retina was analyzed in four of 67.61 × 67.61 μm^2^ fields from the dorsal section of the midperipheral retina in the nasotemporal plane.

Overall, ser-293 P increased both in size and density of phosphorylation status of Cx36 in the myopic retina.

### Dopamine D_1_ Receptor Agonist and Antagonist Can Affect AII AC Coupling in the Mouse Retina

A downregulated dopaminergic synapse pathway was observed in myopic retinas. It is known that AII–AII AC coupling is modulated by dopamine signaling (Hampson et al., 1992; Kothmann et al., 2009). In the current study, quantitative comparison of the tracer coupling pattern of AII ACs with an agonist and antagonist of D_1_R application was performed in the mouse retina.

**FIGURE 8:**
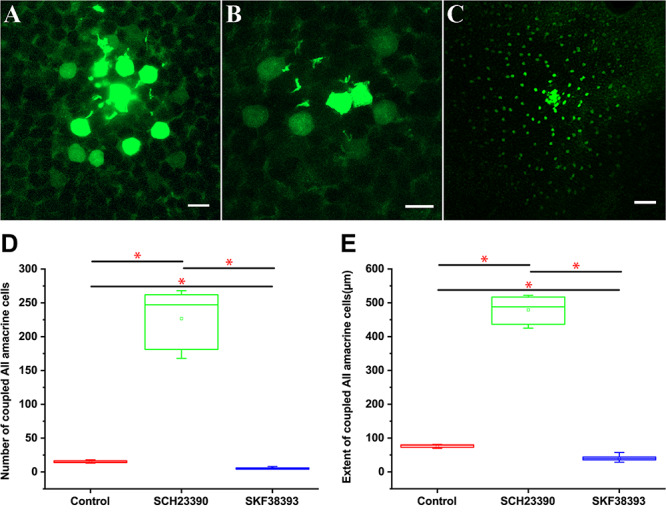
Quantitative comparison of trace coupling between AII amacrine cell changes following application of agonist and antagonist of dopamine D_1_ receptors. **(A)** Flat mount view of a group of tracer coupled AII ACs in the mouse retina following injection of one cell with Neurobiotin. **(B)** D_1_R agonist SKF38393 10 μM reduced the extent of Neurobiotin diffusion in AII AC coupling. **(C)** D_1_R antagonist SCH23390 5 μM dramatically increased tracer coupling of AII ACs. **(A–C)** planes of focus are on the AII cell somata in the proximal inner nuclear layer. Scale bar: **(A,B)** 10 μm, **(C)** 50 μm. **(D)** Box graph showing the difference in the number of coupled AII ACs with D_1_R agonist SKF38393 10 μM and D_1_R antagonist SCH23390 5-μM application. There was a statistically significant difference (asterisk, *P* < 0.01) in the number of coupled AII ACs. **(E)** Box graph showing the difference in the extent of coupled AII ACs somata with the D_1_R agonist SKF38393 10 μM and D_1_R antagonism SCH23390 5-μM application There was a statistically significant difference (asterisk, *P* < 0.01) in the number of coupled AII ACs.

Prior to intracellular injections, retinas were light adapted with photopic white light illumination. AII ACs injected with Neurobiotin displayed a number of coupled neighbors from approximately the first and secondary tier in the control retina group (Figure 8A). Injection of Neurobiotin with activation of D_1_Rs by SKF38393 application (10 μM, *n* = 7) led to a decrease in the extent of Neurobiotin diffusion in AII–AII coupling (Figure 8B) compared with the control (Figure 8A) (*n* = 10). In contrast, application of low concentration D_1_R antagonist SCH23390 (5 μM, *n* = 6) dramatically increased AII–AII coupling (Figure 8C). Quantification of the AII ACs tracer coupling pattern included an overall measurement of cells’ number and size of the tracer-coupled field. Dopamine receptor antagonist (SCH23390, 5 μm) application significantly increased AII ACs coupled cell numbers by Neurobiotin injection from 15 ± 3 (mean ± SD) in the control retina to 226.6 ± 43.2 μm (mean ± SD). At the same time, the extent of the tracer coupled AII ACs rose from 77 ± 8.5 μm^2^ in the control to 479 ± 41.5 μm^2^ in the SCH23390 treated retina. This was in contrast to the effect of D_1_R agonist (SKF38393, 10 μm), in which Neurobiotin-labeled AII cell numbers decreased to (5.4 ± 1.4) and the extent of coupled AII ACs was reduced (40.1 ± 9.1 μm^2^) compared with the control group (Figures 8D,E).

Overall, D_1_R antagonist application significantly increased (*P* < 0.01) in the coupled AII ACs compared with the control group and those with D_1_R agonist application.

## Discussion

It is well established that the retina contains mechanisms to sense the focus of images. The retina plays a large part in governing the emmetropization process by generating signals to regulate eye growth during childhood (Wallman and Winawer, 2004; Pardue et al., 2013).

### Mouse Model of Myopic Retina

Mouse eye growth models provide an excellent means to study refractive development with proven utility for mammalian eye growth and human refractive development. Recent research has shown ON activity tends to inhibit myopia, and OFF activity tends to inhibit development of hyperopia in mice (Chakraborty et al., 2015) and chicken (Crewther and Crewther, 2003). Our research has also shown that defocused images change the signaling of some ON and OFF α RGCs and ON–OFF RGCs in the mouse retina (Pan, 2019; Banerjee et al., 2020). From its already recognized biological effects, retinal signaling is a strong candidate for mediating the retina-to-sclera signaling pathway in refractive development and may be involved in the development of human myopia. Refractive errors less than −5 D of mice after lid suturing confirmed by both OCT and infrared photo refractor measurement were selected for use as the mouse model of myopia. To make the form deprivation model, the presence of cataract and corneal damage (confirmed by infrared photorefraction) should be avoided in mouse myopic model. The FDM mouse model by lid suture method unavoidably influences the light transmission to the eye, which obstructs the intended light exposure. However, the animals are maintained under a 12 h–12 h day–night cycle, and mice have thin thickness of mouse eyelid. The method is unlikely to make prolonged dark adaptation in mouse retina.

### Analysis From scRNA-Seq Indicated Dopamine and Gap Junction Pathways in AII AC Involved in Myopia Development

The cell types involved in myopic development or interaction between these cells’ types in the myopic retina are still not clear. Retinal transcriptome sequencing by traditional population-based genome-wide approaches was not useful in differentiating cell types because all cell types were pooled and cannot be separated and identified. With the development of transcriptome sequencing technology, single-cell transcriptome sequencing, which has been extensively applied in several research areas, can identify each cell type (Subramanian et al., 2005; Darmanis et al., 2015; Haber et al., 2017; Puram et al., 2017). This technique allows all information at RNA level to be obtained. Single-cell RNA sequencing has also been applied in retina research, resulting in 39 cells clusters to be classified in the mouse retina (Macosko et al., 2015). More recently, further cell types—bipolar cells, retinal ganglion cells, and new cell subtypes with biomarkers—have been defined (Shekhar et al., 2016; Rheaume et al., 2018). With the application of current single-cell RNA sequencing techniques, it is possible to identify and acquire pathway information of retinal cells at both the cellular and subcellular levels.

Analysis of the form deprivation-induced myopic retina by single-cell RNA sequencing revealed that dopaminergic synapse and gap junction pathways were downregulated, but Gjd2 mRNA expression in AII ACs was unchanged. In addition, numbers of AII ACs did not differ in the form deprivation-induced myopic retina compared with the fellow eye.

### Gap Junction in AII ACs and Retinal Signaling

Gap junctions play a critical role in intercellular signal communication and information processing in the central nervous system and modulation of visual signals in the retina (Bloomfield and Volgyi, 2009). Recent work indicates that gap junctions are also involved in progressive cell death and aberrant activity seen in various pathological conditions of the retina (Akopian et al., 2014, 2017; Ivanova et al., 2015; O’Brien and Bloomfield, 2018). Because of the characteristics of high plasticity and relation to activity-dependent synaptic input, gap junctions tune retinal microcircuits for processing of visual information under different conditions (Fischer and Stell, 1999; Bloomfield and Volgyi, 2004; Urschel et al., 2006; O’Brien and Bloomfield, 2018; Yadav et al., 2019).

The AII ACs, as a major intersection of rod and cone pathways, segregate signals into ON and OFF pathways in both scotopic and photopic light conditions (Bloomfield and Volgyi, 2004; Basbaum, 2008; Murphy and Rieke, 2008) AII ACs not only contribute to signal averaging and noise reduction under scotopic conditions, but also optimize spatial resolution by “crossover inhibition” (the inhibition of OFF-bipolar and OFF-RGCs by ON-cone bipolar cells via AII ACs) under photopic conditions (Olveczky et al., 2003; Murphy and Rieke, 2008; Arman and Sampath, 2012; Grimes et al., 2015). In the normal retina, gap junctions between ACs and RGCs may attenuate and filter temporal noise to enhance or reduce the probability of firing in coupled RGCs or ACs, thereby modulating RGC output signals to shape visual processing, such as computing local contrast in the circuit (Veruki and Hartveit, 2002; Olveczky et al., 2003).

Connexin36 is the predominant subunit of gap junctions in AII ACs. Glutamate released from bipolar cells activates calmodulin kinase II (CaMKII), which phosphorylates Cx36, promoting gap junction coupling (Kothmann et al., 2012). In this study, Ser293-P increased in density and size in the mouse myopic retina. The defocused image in myopia had a blurred edge and slightly decreased light intensity compared with the focused image. ON RGCs showed more background noise under defocused image projection (Figures 3D,E). The AII AC network is thought to reduce noise and average the signaling (Hartveit and Veruki, 2012). In the defocused experiment, the probability of spikes of both OFF α-RGC and ON RGC showed a significant decrease, which returned totally to normal levels after the image was refocused. Interestingly, the 95% confidence limits were similar in both focused and defocused images, even though background noise increased in ON RGC response in the experiment. It is possible the ACs could have a mechanism to filter out the background noise while keeping the maximal signal of the RGCs. In addition, coupling of AII ACs is also strongly modulated by dopamine, which was confirmed in single-cell RNA sequencing and also involved in myopia development (Zhou et al., 2017). Therefore, more functional Cx36 gap junctions between AII ACs may be needed to adapt in the noisier myopic retina.

### Phosphorylation of Cx36 in AII AC and Myopia

In this study, the major finding was the increased phosphorylation of Cx36 in AII ACs in mouse myopic retinas. The plasticity of gap junctions has diverse roles in the transmission and processing of visual information. The endogenous regulation of gap junctions in AII ACs could be adapted at a certain stage of myopia, leading to increased phosphorylation of Cx36 and coupling to optimize visual processing under the defocused status.

**FIGURE 9:**
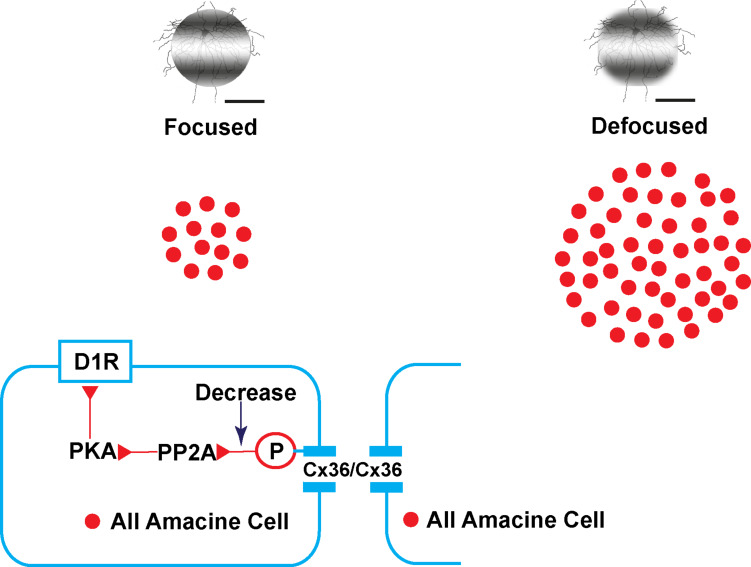
Illustration of the plasticity of gap junctions in AII amacrine cells in myopic and normal retinas. The diagram provides a summary of the intracellular pathways in which the plasticity of retinal gap junctions in AII ACs is affected by dopamine. In the emmetropic retina, images are focused on the retina (left) with dopamine, released from dopaminergic amacrine cells, and bound to D_1_-like receptors (D_1_R) activating cAMP-dependent protein kinase α (PKA) and protein phosphatase 2A (PP2A), which in turn dephosphorylates Cx36, thereby causing reduced gap junction conductance. In contrast, decreased dopamine increases the conductance of Cx36 in AII amacrine cells by increasing phosphorylation in the defocused status of the myopic retina (right).

By promoting phase locking of oscillations, gap junctions contribute to accurate visual signaling transport to downstream networks (Pernelle et al., 2018). It can be hypothesized that defocused images in myopia are noisy and carry much more visual information downstream than focused images. Therefore, more coupling of AII ACs or functional gap junctions (Cx36) may be needed to filter noise. It has also been shown that phosphorylation could be adjusted to a homogeneously high level with pharmacological agents (Kothmann et al., 2007, 2012). Dopamine acts as an important neurotransmitter in the retina and mediates retinal development, visual signaling, and refractive development. In this experiment, dopamine D_1_ receptor agonist and antagonist application induced changes of trace coupling between AII ACs. It may be hypothesized that the mechanism of plasticity of phosphorylation of Cx36 in AII ACs in emmetropic and myopic retina involves the following (Figure 9): in the emmetropic retina, dopamine release from dopaminergic ACs combined with the D_1_R. Dopamine receptor-driven uncoupling of the AII network results from PKA activation of PP2A and subsequent dephosphorylation of Cx36, to decrease the conductance in AII ACs with focused images in the emmetropic retina. The defocused image generates noisier signaling in the myopic retina, leading to a need for increased functional phosphorylation of the Cx36 in AII AC coupling to filter out the noise. At a certain stage of myopia development, the dopamine level decreases to adapt to the defocused status (Feldkaemper and Schaeffel, 2013). Through a combination of plasticity of their electrical and chemical synapses, AII ACs actively adapt differently to focus and defocus states.

## Conclusion

In conclusion, this study expands the understanding of the gap junction and suggests new potential targets for increasing gap junction activity between AII ACs to restore vision acuity in myopia prevention. In the future, further studies are needed to distinguish decreased phosphorylation of Cx36 related to normal physiology (focused image) or the maladaptive changes (focused image) that occur as myopia progresses. It is also necessary to perform further research to clarify the complex etiology and nature of physiological remodeling in myopia.

### Limitations of the Study

The results demonstrated that increased phosphorylated Cx36 could indicate increased gap junction coupling of AII ACs in the mouse myopic retina. The evidence provided in the research is correlative here. To test whether coupling of AII ACs also increased in the myopic retina, Neurobiotin injection was performed in our preliminary experiment. Considering the light will affect the coupling of AII ACs dramatically (Bloomfield and Volgyi, 2004), we did Neurobiotin injection into AII ACs of around −3-D myopic mouse model under both dark and dim light adaptation conditions (Bloomfield and Volgyi, 2009) in the preliminary experiment. However, we did not find the convincing result to show that the coupling of AII ACs elevated in myopic retina in the trial. The following possible reasons might be involved: (1) the coupling of AII ACs change indicated by Neurobiotin injection method might relate with refractive power of myopic retina. For example, retina with lower than minus 5 D might show the increased coupling of AII ACs. (2) Gap junctions are highly plastic and quickly regulated by light-activated neuromodulators or image (O’Brien and Bloomfield, 2018). Compared with coupling change of AII ACs induced by myopia, the dark and/or light adaptation might contribute more effects on coupling of AII ACs. Then the change induced by myopia was masked under the different light adaptation by Neurobiotin injection method. Thus, a direct evidence from Neurobiotin injection method to show the increased coupling of AII ACs in myopic retina is difficult because of the different light adaptation conditions, myopic refractive powers, and variation of dopamine levels. The patch recording of dual or multiple AII ACs under the focused/defocused images or between myopic/normal retina might show the signaling change in AII ACs. However, there still were technique and experimental design challenges here. In addition, this result may not be able to be translated to explain myopia in humans because of differences in anatomy and development of the eye. The phosphorylation status of Cx36 in the mouse myopia model was observed on postnatal day 56, so its long-term effects in myopic retina are not known. Nevertheless, this study illustrates adaptations to adjust to the noise signaling status in the myopic retina.

## Data Availability Statement

The datasets generated for this study are available on request to the corresponding author.

## Ethics Statement

The animal study was reviewed and approved by the Animal Subjects Ethics Sub-Committee (ASESC) of the Hong Kong Polytechnic University and the Animal Care and Ethics Committee at Wenzhou Medical University.

## Author Contributions

FP, SB, QW, GT, CS, and FZ contributed to the acquisition, analysis, and interpretation of data. YF, DT, and C-HT contributed to the collection of part of the data. XZ and FP contributed to the conception and design of the work and contributed to the drafting the manuscript.

## Conflict of Interest

The authors declare that the research was conducted in the absence of any commercial or financial relationships that could be construed as a potential conflict of interest.

## Supplementary Material

FIGURE S1 Dopaminergic synapse pathway of AII amacrine cell clusters. Dopaminergic synapse pathway was downregulated in the treated eyes compared with fellow eyes. The genes marked with light blue are leading edge genes of the Gene Set Enrichment Analysis (GSEA) enrichment. These blue labeled leading edge genes^+^ in AII/AII amacrine cells account for more than 10% of the total. The figure cited from KEGG (Kyoto Encyclopedia of Genes and Genomes) and copyright was permitted.

FIGURE S2 Gap junction pathway of AII amacrine cell clusters. The gap junction pathway was downregulated in treated eyes compared with fellow eyes. The genes marked with light blue are leading edge genes of GSEA enrichment, leading edge genes^+^ AII/AII amacrine cells > 0.1 in fellow eyes. The figure cited from KEGG (Kyoto Encyclopedia of Genes and Genomes) and copyright was permitted.

TABLE S1 Details of gene expression of leading edge subset genes involved in gap junction and dopamine pathways.
