# Supplementary figures and images for: Increased Connexin36 Phosphorylation in AII Amacrine Cell Coupling of the Mouse Myopic Retina
Source: Front Cell Neurosci. 2020 Jun 1;14:124. doi: 10.3389/fncel.2020.00124 (PMC7278884; doi:10.3389/fncel.2020.00124)

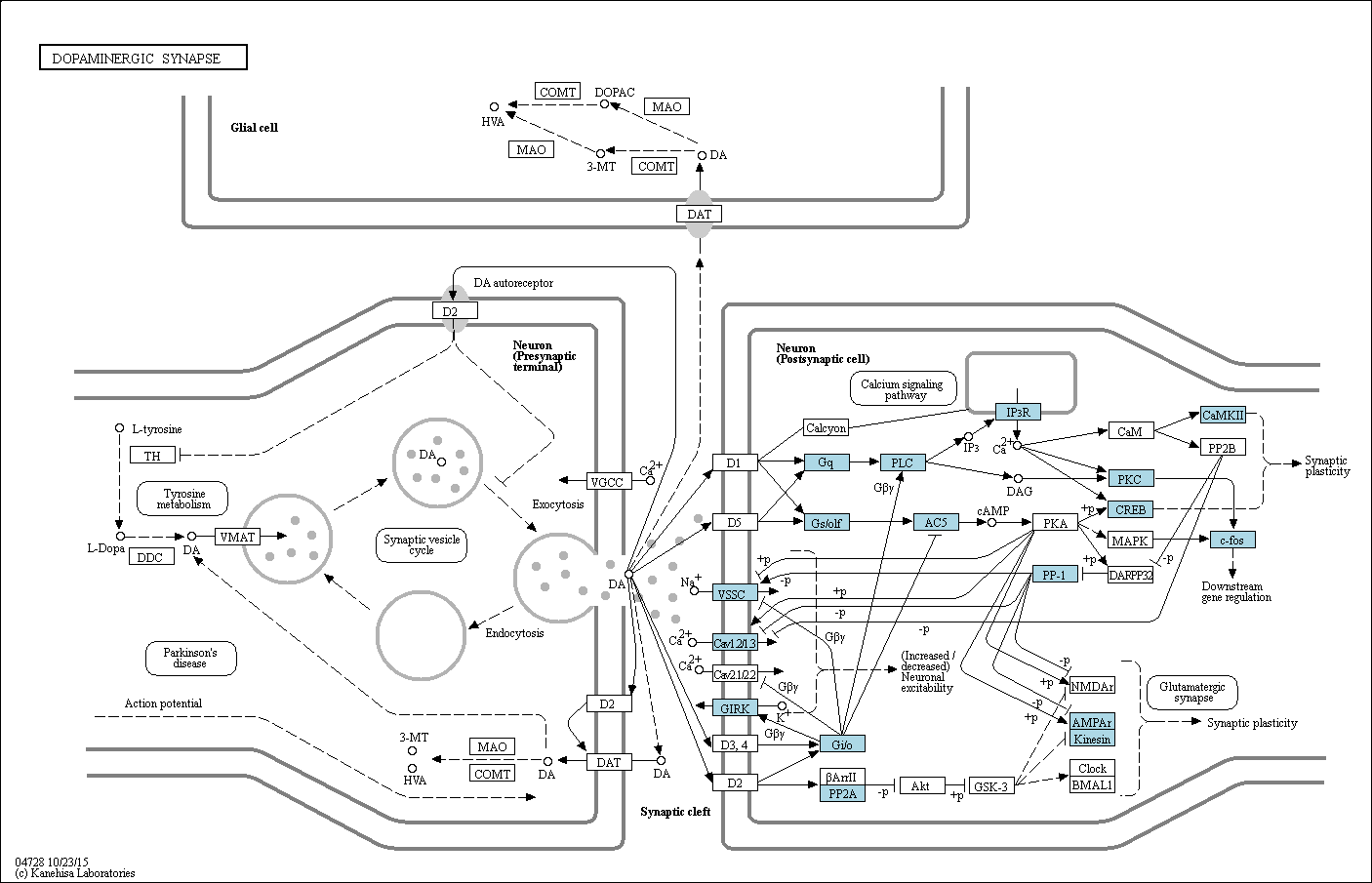

Supplement: FIGURE S1 — Dopaminergic synapse pathway of AII amacrine cell clusters. Dopaminergic synapse pathway was downregulated in the treated eyes compared with fellow eyes. The genes marked with light blue are leading edge genes of the Gene Set Enrichment Analysis (GSEA) enrichment. These blue labeled leading edge genes+ in AII/AII amacrine cells account for more than 10% of the total. The figure cited from KEGG (Kyoto Encyclopedia of Genes and Genomes) and copyright was permitted. [file Image_1.TIF]

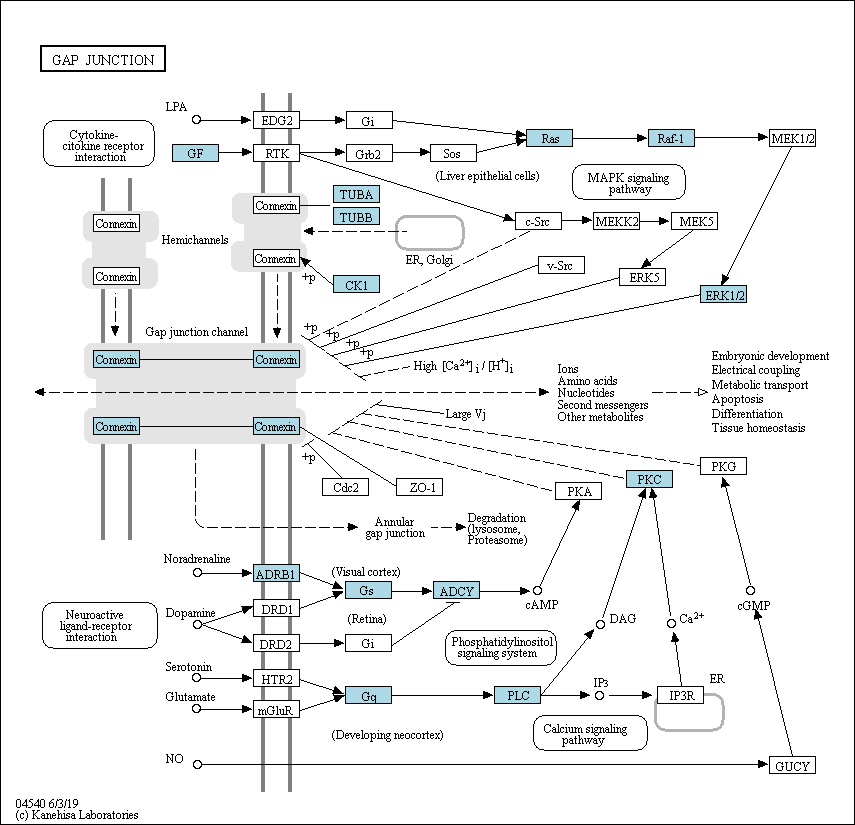

Supplement: FIGURE S2 — Gap junction pathway of AII amacrine cell clusters. The gap junction pathway was downregulated in treated eyes compared with fellow eyes. The genes marked with light blue are leading edge genes of GSEA enrichment, leading edge genes+ AII/AII amacrine cells > 0.1 in fellow eyes. The figure cited from KEGG (Kyoto Encyclopedia of Genes and Genomes) and copyright was permitted. [file Image_2.TIF]
